# Microvascular transposition using Teflon sling technique

**DOI:** 10.3171/2020.6.FOCVID2040

**Published:** 2020-10-01

**Authors:** Mitchell W. Couldwell, Vance Mortimer, AS, William T. Couldwell

**Affiliations:** Department of Neurosurgery, Clinical Neurosciences Center, University of Utah, Salt Lake City, Utah

**Keywords:** microvascular transposition, vascular conflict syndrome, cranial nerve pain, sling technique

## Abstract

Microvascular decompression is a well-established technique used to relieve abnormal vascular compression of cranial nerves and associated pain. Here the authors describe three cases in which a sling technique was used in the treatment of cranial nerve pain syndromes: trigeminal neuralgia with predominant V2 distribution, hemifacial spasm, and geniculate neuralgia and right-sided ear pain. In each case, the artery was mobilized from the nerve and tethered with a sling. All three patients had reduction of symptoms within 6 weeks.

The video can be found here: https://youtu.be/iM7gukvPz6E

## Transcript

This case demonstrates the technique that we use for microvascular transposition for vascular conflict syndromes.

**0:28 Microvascular Decompression for Treatment of Trigeminal Neuralgia.** This is a 60-year-old woman with left-sided trigeminal neuralgia, primarily in the V2 distribution. You can see there is a conflict of a vessel at the root entry zone of the fifth nerve on the left side.^1^ She is placed in the lateral position. For this case, we’ll monitor motor evoked potentials, somatosensory evoked potentials, facial nerve function, and auditory brainstem responses. We place a very targeted surgical incision, which is S-shaped, over the trigeminal hole, which is made here and is approximately the size of a dime.

We open up the arachnoid at the petrotentorial junction and identify the root entry zone of the fifth nerve. We see a vein lying on the fifth nerve on this inferior side, and then we dissect superiorly to the nerve and identify a conflicting superior cerebellar branch as well. We’ll free up the superior cerebellar branch from its arachnoid investment and move the artery away from the root entry zone of the fifth nerve.

We use Teflon tape as a sling, and then sling the artery superiorly toward the tentorium and suture this in place with 8.0 suture.^2^ We use one or two sutures per sling to hold the artery away. The nerve is then massaged to afford immediate pain relief, and fibrin glue is used to help secure the sling in place. The fascial graft that was obtained with the opening is sutured in place to provide a watertight dural closure, and fibrin glue is placed. A burr-hole cover is then used to cover up the small opening. The scalp is closed in successive layers. The patient had immediate relief of her facial pain.

**2:58 Microvascular Decompression for Treatment of Hemifacial Spasm.** This case demonstrates a right-sided microvascular transposition for hemifacial spasm in a 52-year-old woman who had failed medical therapy with Botox after many years.^3,4^ She had obvious conflict at the root exit zone of the seventh nerve on the right side. We’ll use the lateral position once again and monitor the motor evoked potentials, somatosensory evoked potentials, facial nerve monitoring, auditory brainstem monitoring, as well as a Medtronic NIM tube to measure EMG monitoring of the 10th nerve. We’ll plan an opening here behind the mastoid and perform a roughly 2-cm opening and identify the 9th–10th–11th complex and the 7th–8th nerve complex.

Her surgery is targeted between the 9th, 10th, and 11th nerves and the 7th–8th complex. We see a branch of ICA, which has been compressing the seventh nerve, and we mobilize this and then place a sling around the ICA artery, and then mobilize it over toward the posterior petrous bone. We then use our 8.0 suture to again suture to the dura of the petrous bone and transpose the artery away so nothing is touching the root exit zone of the seventh nerve. We ensure that there is nothing touching the nerve, place fibrin glue on the complex, and then proceed with closure. Following surgery, she had gradual decrease of her hemifacial spasm that completely abolished over the next 6 weeks.

**4:57 Microvascular Decompression for Treatment of Geniculate Neuralgia.** This last video demonstrates the treatment of geniculate neuralgia in a 45-year-old woman with right ear pain. Her imaging performed demonstrates a conflict of the seventh and eighth nerve and presumably the nervus intermedius on the right side. After positioning, we’ll place electrodes to measure facial nerve function, auditory brainstem responses, and again a NIM tube to measure 10th nerve function. A small hole is planned overlying the 7th and 8th nerves and the 9th, 10th, and 11th nerves.

We open up here over 7 and 8 on the left and 9, 10, and 11 on the right. The first step is to cut the ninth nerve, and then we explore the seventh and eighth nerve complex, and we can see this is a very difficult situation here. We’ve got the ICA branch running between 7 and 8, limiting our microvascular decompression possibilities. We identify the nervus intermedius and cut this.^5,6^ We then mobilize the small ICA branch, and we’ll place a sling to the posterior petrous bone in an effort to decrease the microvascular conflict. Fibrin glue is used to hold the sling to the petrous bone. The wound is then closed using a fascia graft that was obtained at opening, and a large burr-hole cover was used to cover the bone opening. The patient had improvement in her ear pain that was immediate.

## Author Contributions

Primary surgeon: WT Couldwell. Editing and drafting the video and abstract: all authors. Critically revising the work: WT Couldwell. Reviewed submitted version of the work: WT Couldwell. Approved the final version of the work on behalf of all authors: WT Couldwell.
